# Does a short-term intervention promote mental and general health among young adults? – An evaluation of counselling

**DOI:** 10.1186/1471-2458-7-319

**Published:** 2007-11-08

**Authors:** Regina Winzer, Agneta Bergsten Brucefors

**Affiliations:** 1 Swedish National Institute of Public Health, SE-831 40 Östersund, Sweden; 2 Karolinska University Hospital Huddinge and Karolinska Institutet, SE-141 86 Stockholm, Sweden

## Abstract

**Background:**

Since 1988, self-reported mental health problems in Sweden have increased more among young people than in any other age group. Young adults aged 18 – 29 with minor mental health problems were welcomed to four (at most) counselling sessions led by psychotherapists. The present study aimed to evaluate the method's appropriateness and usefulness.

**Methods:**

The study population was recruited consecutively during six months (N = 74) and consisted of 59 women and 15 men. Fifty-one, 46 women and five men, met the criterion for a *personal semi-structured interview *three months post intervention. Self-assessed health data were collected on three occasions using the General Health Questionnaire (GHQ-12), Pearlin's Personal Mastery Scale and two items from the Swedish Living Conditions Surveys. Thirteen women and six men were not statistically assessed due to incomplete data, but were *interviewed by telephone*. Four men refused to be interviewed and became *dropouts*.

**Results:**

The largest group of the study population had long been troubled by their problem(s): 43 percent for over three years and 28 percent for over one year. Among those *personally interviewed*, 76 percent reported psychological distress (> 3 GHQ points) before the counselling. After the counselling, GHQ-12 distress decreased by 50 percent while mastery and perceived health status increased significantly. A majority experienced an improved life situation, found out something new about themselves and could make use of the sessions afterwards. Personal participant session contentment was about 70 percent and all counsellees would recommend the intervention to a friend. Those *interviewed by telephone *were not statistically assessed due to incomplete health data. Their personal contentment was just under 50 percent, though all except one would recommend the counselling to a friend. Their expectations of the intervention were more result-orientated compared to the more process-directed personally-interviewed group.

**Conclusion:**

This evaluation shows a clear improvement in self-rated mental and general health, mastery and control in the group completing the study agreement. The intervention seems to be effective for young adults with minor mental health problems, but due to the skewed gender-distribution it is unclear if the method is appropriate for men. After the proposed internal quality improvements, this short-term counselling could enhance mental and general health among young people.

## Background

### Mental health data

Several studies indicate a deterioration in mental health among young people over the past 50 years [1,2]. In the UK, an analysis of three studies shows a rise in emotional problems in young females and males over the study period 1986 – 1999[3]. However, data from the US provide no evidence for a rise in the problems of children and adolescents[4].

In Sweden ten percent of children and adolescents are judged to have mental health problems. They suffer from depression, anxiety or disorders related to aggressive behaviour, have difficulty concentrating; or have eating disorders. Another five-to-ten percent of children and young people have minor forms of mental health problems. The starting age for depressive disorders has decreased and more young people are suffering adverse effects of depression[5].

The prevalence of suicide in Sweden has declined by 30 percent for the total population over the past 20 years, but less so for the 15 – 24 age-group, where it is the second most important cause of death[6].

Self-reported health is a common self-assessment measure. It has a predictive value for future illness and death[7,8]. Swedish national surveys show that self-reported symptoms of nervousness, anxiety, sleeping problems and tiredness have increased for the total population. However, the largest increase has been in the younger age groups, where e.g. adults of 18 – 29 years doubled and in some cases tripled their symptom ratings between 1988 and 2002. In 1988/89, 4.6 percent of young men reported that they felt nervousness and/or anxiety and in 2002 this figure had increased to 14.9 percent. For women, the corresponding increase was from 8.9 percent to 28.2 percent, while sleeping problems rose among men from 7.1 percent to 20.0 and among women from 10.5 percent to 27.4 percent. Tiredness tripled for young men from 3.4 percent to 10.3 percent and doubled for young women from 8.4 percent to 16.4 percent[9]. Other international, national and regional surveys for the past decade show the same tendencies [10-12].

The prescription of hypnotic drugs for all age groups increased by more than 100 percent from 1991 to 2004. Young women in the 15 – 19 age group had the largest increase, i.e. their purchased prescriptions rose from 0.2 to 1.8 defined daily doses (DDD)/1000 inhabitants a day. Women aged 20 – 24 years increased their purchases from 1 to 7.5 DDD/1000 inhabitants a day. The most dramatic rise is seen for prescription of antidepressant drugs, where young women aged 20 – 24 increased their purchase from 2 to 45 DDD/1000 inhabitants a day between 1991 and 2004[13].

The Swedish national action plan for the health-care system emphasizes the need to offer young people adequate help in the early stages of mental health problems. The plan also points out the need for greater cooperation in outpatient care for adults and young people. In recent years, several county councils have established special counselling units with easily accessible help for those aged 16 – 24 years.

### A mental health offer for young people

In 1999, five counselling psychotherapists from the St. Lukas Foundation in Stockholm started a counselling programme for young adults aged 18 – 29 years with minor mental health problems, e.g. nervousness, worry and sleeping problems, but not psychiatric diagnoses. The therapists each had 15 – 20 years experience of psychodynamic psychotherapy with young and adult people. The target group was recruited through advertisements in a free magazine and clients were invited to attend four counselling sessions after a wait of no longer than two weeks. No client was refused, but a few were referred to other care providers. The National Institute of Public Health sponsored the programme entitled Four-sessions-at-most, and the cost to the client of each session was low for Swedish circumstances, about 7 GBP.

### The counselling method

The counselling method employed was developed at the Young People's Counselling Service, Tavistock Clinic, London as an offer for the "cautious and the curious; cautious about commitment but curious about themselves [14]." The method has proved applicable and useful for young adults in college settings whose problems have not become permanent[15].

This concept of brief intervention has been practised in Stockholm since 1993 at the Institute of Psychotherapy, which introduced the present counselling technique at St. Lukas. The criteria for acceptance are that the client should:

- be actively seeking help,

- be prepared to understand his/her own participation concerning the actual problem,

- be aware of the relatively short-term nature of the contact and accept the limitation of four sessions.

And the therapist should

- focus on the counsellee's actual situation and difficulties,

- activate the counsellee's ability to reflect on her/his situation,

- try to catch the core problem emerging from the counsellee's behaviour during the meeting,

- reduce the counsellee's regression by forming links with external reality.

#### Aim of the present study

Since the method had been in use for a period, it needed to be evaluated. The overall aim of the present evaluation was to study the appropriateness of the method of brief intervention for public health settings and for preventing mental illness. There was also an interest in whether a short contact of four, sometimes only three, meetings could help young people to solve their problems.

### Objectives

The present objectives were to:

- study whether four sessions at most in a short-term counselling programme do affect self-rated health in young adults

- analyse changes in self-rated mental and general health between before and after the sessions, and

- assess the young adult's positive and negative experience of the sessions.

## Methods

### Study groups

The study population was recruited consecutively over six months. Altogether, data were collected from 74 persons.

A) The *personally interviewed group*, 51 persons, followed the procedure as agreed: they completed baseline, attended one to four sessions, completed the first follow-up questionnaire in connection with the last session and thus met the criterion for a personal interview three months after the last session. Together with the interview they completed their second follow-up questionnaire.

B) The *telephone-interviewed group*, 19 subjects, completed baseline, attended one to four sessions, but did not complete the first follow-up questionnaire and thus did not meet the criterion for a personal interview. They were interviewed by telephone at a two-month follow-up.

C) Four subjects completed baseline, attended one to four sessions, but did not complete the follow-up questionnaire and did not answer an invitation to be interviewed by telephone. They formed a *dropout group*.

### Instruments

#### The personal questionnaire

It was important to get information about the participant's background, what problems they wanted to discuss, how long they had been experiencing problems, and what their expectations of the sessions were. Applicants filled in the personal questionnaire only once, in connection with the self-evaluation completed on their first visit to St. Lukas.

#### The GHQ-12, self-evaluation

Goldberg's General Health Questionnaire [16] is a commonly-used instrument in health surveys. The questionnaire is intended as a short, easily administered screening instrument for identifying individuals with minor mental health problems. It is now often used in population- and work-environment studies to estimate mental health among groups and individuals. The GHQ-12 may be understood as a measure of one dimension of mental distress. The scale focuses on the here-and-now situation: when answering the questions, the individual compares the present with how things used to be. The grading in this study is by GHQ points (0, 0, 1, 1). The GHQ-12 has been validated for Swedish conditions and has proved very useful in measuring mental health among young people[17]. In Europe, it has been used in e.g. multi-centre studies of young students at colleges of dentistry[18], and in studies of counselling at the University of Sheffield [19]. In Sweden the GHQ-12 is used by the National Institute of Public Health and by several county councils in their health investigations. The cut-off for GHQ points indicating minor health problems is usually > 3 and the same value is used in this evaluation.

#### Pearlin's Personal Mastery Scale, self-evaluation

Mastery of one's life and one's life situation is reportedly a protective factor against unhealthy stress. Such mastery is also an important ability when changes in life occur [20]. The Mastery Scale consists of seven items[21]. Five measure mastery-control and two measure mastery-action. The items are answered on a four-point scale of agreement: from "do not agree at all" to "fully agree". A high score on the Mastery Scale indicates a high degree of mastery (7 – 28 points). The Scale has been used in Sweden by e.g. Anderzén[22].

#### General Health, two self-evaluating items

Two self-evaluation items are used by Statistics Sweden in population studies, the Living Conditions Surveys (ULF)[23]. The first item, "How is your general health?", has alternative answers 'very good', 'good', 'reasonable', 'bad' and 'very bad.' The second is "How is your general health, compared to that of other people of your own age?" This item has three alternatives: 'better', 'about the same' and 'worse'. In ULF, the first question is asked every year and the second every sixth year.

All the questionnaires were applied on three occasions: before the intervention took place (baseline), immediately after the last session (post-test 1), and three months after the last session, immediately before the personal follow-up interview (post-test 2).

#### The personal follow-up interview

The aim of the follow-up interview was to obtain knowledge about what the counsellees understood, and how they felt, about the sessions three months later. Thirteen questions about the advantages and disadvantages of the meetings with the psychotherapist were developed. Each interview took 40 – 50 minutes and was tape-recorded. Following an interpretive research approach, the answers were read, coded, categorized, classified into different themes and interpreted [24].

#### The telephone interview

The aim of the telephone interview was to find out whether the meetings with the therapist had been completed and to form an impression of how the counsellee assessed them. For this reason, six core questions of the 13 from the follow-up interview were asked during a brief telephone call.

In the present study we mainly present data from the *personally-interviewed group *as we could follow their progress systematically. Data from the *group interviewed by telephone *are used from both their personal questionnaire and the telephone interview.

### Procedure

The study was approved by the Ethical Review Board at Karolinska Institutet, Stockholm, Sweden. The evaluation was carried out as follows. The secretary at St. Lukas in Stockholm, who answered the first telephone call, asked the applicants whether they wanted to take part in the evaluation and whether they accepted the condition that they had to answer the questionnaires on three occasions. Participants who accepted were invited to meet a psychotherapist and the secretary explained how the evaluation was to be performed. All applicants except one accepted inclusion in the study. Before the first meeting, the participants received the following information and instruments by post:

- a handout giving a background and informing about the aims of the evaluation,

- the personal questionnaire,

- the three self-rated screening questionnaires GHQ-12, Pearlin's Personal Mastery Scale and the General Health, two self-evaluating items.

The participants were asked to fill in the questionnaires and bring them in a sealed envelope to their first meeting. They were also told they would receive four cinema tickets after completing the final interview.

Statistical analyses were performed using SPSS, versions 11, 12 and 15. Differences between groups were tested with the non-parametric tests Fisher's exact test, the Wilcoxon Matched Pairs Signed Ranks Test, the Cochran, McNemar and Friedman tests. As a measure of reliability for the GHQ-12 and Mastery Scale, Cronbach's alpha for internal consistency was computed.

## Results

### Demographic breakdown

The original study population consisted of 74 persons, 59 women and 15 men. According to the inclusion criteria they were divided into a personally-interviewed group, 46 women and five men, and an interviewed-by-telephone group, 13 women and six men. The differing gender distribution in these two groups (Fisher's exact test: p < .05) implies that the women fulfilled the study agreement more than the men did. Additionally, the drop-out group consisted of only men (N = 4).

The mean age of those personally interviewed was 24.5 years and of those interviewed by telephone 23.2 years, a non-significant difference.

As most of the young population had not completed their education it was less useful to specify them into narrow professional categories. Instead broader ones were used. Among those personally interviewed who answered the question about occupation/employment (N = 50) the largest group among women consisted of students and academic professionals, 17 subjects in each. Three men were students and two worked in personal care and service (Table 1). The corresponding group interviewed by telephone (N = 17) was dominated by those working in personal care and service, e.g. chemist's assistants, recreation leaders and shop assistants. In contrast to the former group only one woman had an academic profession and in both groups no male worked in an academic field.

**Table 1:** The counsellees' profession/employment (N = 67)

	Female Personally interviewed	Male Personally interviewed	Female Interviewed by telephone	Male Interviewed by telephone	Total
Student	17	3	3	2	25
Academic profession	17	0	1	0	18
Personal care and service	6	2	6	2	16
Design and crafts	3	0	1	0	4
Unemployed	1	0	0	0	1
Other	1	0	1	1	3
Total	45	5	12	5	67

The groups were ethnically highly homogenous; three persons were of non-Scandinavian ethnicity.

### Problem areas

Seventy counsellees, 56 women and 14 men, answered the question concerning what they wanted to talk about (Table 2). They often mentioned more than one area, causing the answers to overlap somewhat. Relationships constituted the major problem areas among women, 17 reporting problems in their relationships with their partner, family and friends. Also more diffuse problems such as "everything", "me and my sorrows" and "life and depression, feeling low", and "feeling bad" were often mentioned.

**Table 2:** Counsellees' description of problem areas (N = 70). Several alternatives possible.

	Female % (N = 56)	Male % (N = 14)	p^1^
Relations	30% (17)	21% (3)	n.s.
"Everything", "life", etc	21% (12)	43% (6)	<.10
Depression, "feeling low", etc	23% (13)	14% (2)	n.s.
Difficulties keeping within limits	9% (5)	14% (2)	n.s.
Stress and insomnia	11% (6)	7% (1)	n.s.
Identity problems, low self-esteem	11% (6)	0% (0)	n.s.
Work or studies	9% (5)	0% (0)	n.s.
Separation, illness, death	7% (4)	7% (1)	n.s.

^1^Fischer's exact probability test

### Duration of the problem experienced

In general the counsellees had had their problems for a long time (Table 3). In the study population answering the question about duration (N = 64) the largest group, 42 percent, had been troubled for over three years and 28 percent for more than one year. Among those *personally interviewed *answering this question (N = 45), the largest group had had problems over three years (47 percent). Especially young women tended to have experienced their problems for longer. In contrast, the group *interviewed by telephone *(N = 19) had experienced their problems for a shorter time, women again comprising most of the group with problems longer than three years.

**Table 3:** Duration of the problem experienced (N = 64)

	Female Personally interviewed	Male Personally interviewed	Female Interviewed by telephone	Male Interviewed by telephone	Total
From one week to a few weeks	0	0	2	0	2
1–5 months	4	1	0	1	6
6–12 months	5	1	3	2	11
>12 months	12	1	3	2	18
>3 years	20	1	5	1	27
Total	41	4	13	6	64

### Expectations

Of 74 counsellees, 69 wrote about their expectations from the counselling, 55 women and 14 men (Table 4). Twenty expected to be better able to handle problems and conditions afterwards. Fourteen, 13 women and one man, were seeking a new approach to their lives and to formulate fresh patterns of thinking. Thirteen wanted an explanation of their conditions and problems.

**Table 4:** Counsellees' expectations (N = 69). Several alternatives possible.

	Female % (N = 55)	Male % (N = 14)	p^1^
Be better able to handle problems and conditions	27% (15)	36% (5)	n.s.
New approach to life, fresh patterns of thinking	24% (13)	7% (1)	n.s.
Insight/knowing myself better	20% (11)	7% (1)	n.s.
Explanation of conditions and problems	15% (8)	36% (5)	<.10
Feeling better	5% (3)	29% (4)	<.05
Relief	7% (4)	7% (1)	n.s.
Further psychotherapy in future	5% (3)	7% (1)	n.s.
Low or no expectations	5% (3)	21% (3)	<.10
Do not know	5% (3)	7% (1)	n.s.

^1^Fischer's exact probability test

Among those personally interviewed answering the question (N = 49) the two categories dominated: "to be better able to handle problems and conditions", (12 females and 2 males), and "to get a new approach to life, and fresh patterns of thinking" (12 females and 1 male).

Those in the group interviewed by telephone responding to the question (N = 18) the dominating categories consisted of "to get an explanation of conditions and problems"(5 females and 3 males) and "feeling better" (2 females and 2 males).

### Self-rated mental and general health in the personally interviewed group

#### GHQ-12

**Figure 1:**
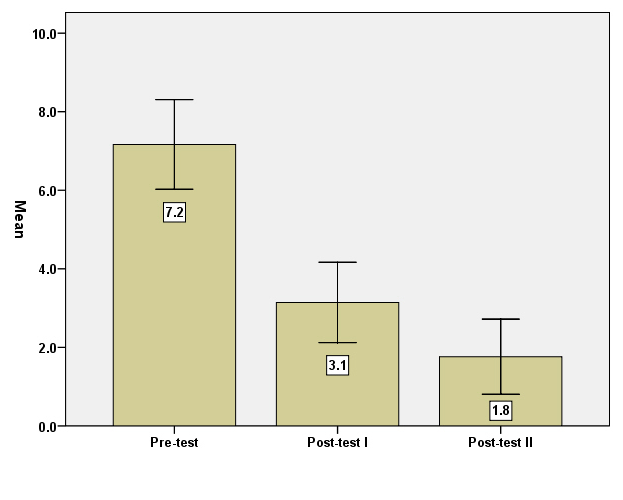
GHQ-12 points, means, before = baseline (I), immediately after (II) and three months after (III) the intervention. Error Bars: +/- 1.96 SE.

Mental health status was measured with the GHQ-12. The average level was poor on the first occasion (Figure 1): the higher the score, the worse the mental health. Cronbach's alpha was r = .88 for the items in the GHQ-12.

The differences in GHQ points between baseline and post-test 1 were significant (p < .001), as were those between post-tests 1 and 2 (p < .05). The same results were gained when only women were considered.

**Figure 2:**
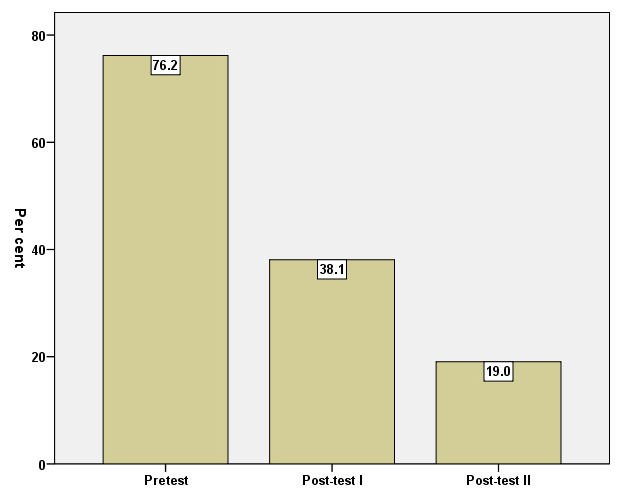
Percentage of caseness according to GHQ-12 (cut-off >3) before = baseline (I), immediately after (II) and three months after (III) the intervention.

According to the GHQ manual [16], caseness for psychological distress (morbidity) may exist at >3 GHQ scores. The amount of caseness at the three measuring points is shown in Figure 2.

The decrease between baseline and post-test 1 was statistically significant (p < .001). The decrease between post-test 1 and post-test 2 was not significant (p > .05).

#### Mastery

**Figure 3:**
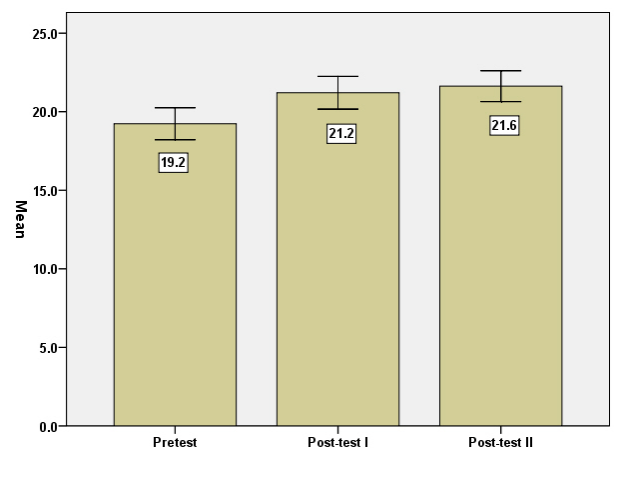
Mastery Scale, means, before = baseline (I), immediately after (II) and three months after (III) the intervention. Error Bars: +/- 1.96 SE.

The Mastery Scale mean increased significantly between baseline and post-test 1 (p < .01). No further improvement was found three months later (Figure 3). Cronbach's alpha was r = .61.

#### General health

**Figure 4:**
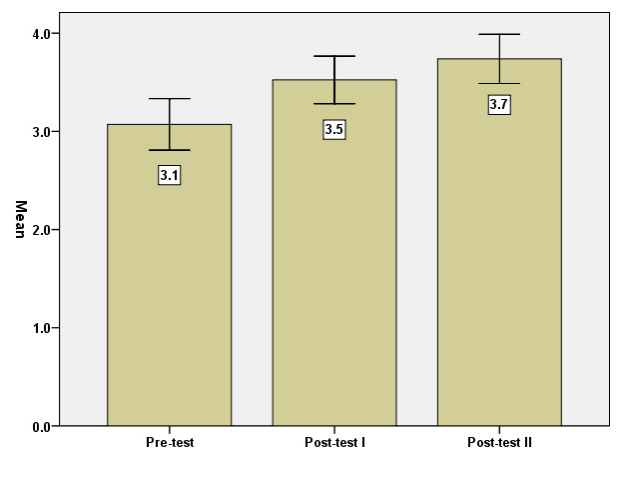
General health, means, before = baseline (I), immediately after (II) and three months after (III) the intervention. Error Bars: +/- 1.96 SE.

Answers to the question "How is your general health?" show that the mean rose significantly between baseline-test and post-test 1 (p < .01) (Figure 4).

Another aspect of this question is the proportion who assessed their health as bad/very bad on the three occasions. This proportion decreased significantly (p < .01) from 27 percent to 7 percent between baseline-test and post-test 1. No further decrease was shown three months later.

#### General health compared with coevals'

**Figure 5:**
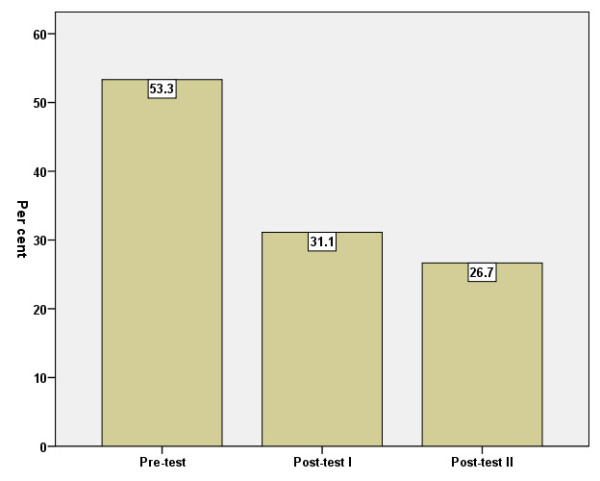
Perceived own health as worse than coevals', per cent, before = baseline (I), immediately after (II) and three months after (III) the intervention. Error Bars: +/- 1.96 SE.

The responses to the second question, "How is your general health compared to other people's of your own age?", show that 53 percent of the personally-interviewed group assessed their mental health as worse than that of other people of the same age (Figure 5).

The decrease between baseline and post-test 1 was significant (p < .05) but not that between post-test 1 and post-test 2.

### The personal follow-up interview (N = 51)

#### Satisfied and very satisfied with the intervention

Five interview questions elicited comments on individual satisfaction with the intervention and were used to dichotomise those who were content or not content, respectively, with the counselling. A majority of the personally interviewed, 32 women and 3 men, were satisfied or very satisfied with their sessions. The most frequent statements (Table 5) among them were *having found out something new about themselves or having gained new insight *and *making use of the sessions afterwards*:

**Table 5:** Most frequent statements by counsellees personally interviewed who were satisfied or very satisfied with the intervention (N = 35). Several alternatives possible.

	Female	Male	Total
Found something new about myself/new insight	29	3	32
Could make use of the sessions	25	2	27
Felt understood by the counsellor	24	3	27
New approach to life, fresh patterns of thinking	24	1	25
Improved life situation compared to three months earlier	23	2	25
Obtained what I wanted	20	2	22
Be better able to handle problems and conditions	12	1	13
Relief to talk to a neutral person	11	0	11

"I became aware of my huge need to be in control. Now afterwards I sometimes recognise this feeling and can tell myself: now you are back in your old manner, losing control and feeling bad. This is a great understanding and I am now working on it myself." (Woman)

*Getting a new approach to life and/or fresh patterns of thinking *was another aspect frequently mentioned by women, and once by a man:

"I saw my problem from a new angle, realised that my part of it was not as big as I thought before." (Man)

#### Doubtful, less satisfied or very dissatisfied with the intervention

However, 16 counsellees, or 30 percent, were less satisfied with the intervention. This group of people who were doubtful, less satisfied or very dissatisfied with the intervention consisted of 14 women and two men. Their criticism (Table 6) ranged from the psychotherapist's attitude and way of relating (too passive, insufficiently committed, lack of feed-back) to the method's time limitation and the St. Lukas payment routines after the interviews:

**Table 6:** Most frequent statements by counsellees personally interviewed who were doubtful, less satisfied or very unsatisfied with the intervention (N = 16). Several alternatives possible.

	Female	Male	Total
Felt no good contact with counsellor	10	2	12
Four sessions are not enough	8	0	8
Counsellor was too passive/gave little feed-back	7	1	8
Felt misunderstood by counsellor	2	1	3
Counsellor gave no advice	0	2	2
The sessions were too short	2	0	2
Unsatisfactory payment routines	3	0	3

"Forty-five minutes was too short a time. We'd hardly started the session before the counsellor looked at her watch and ended it. I talked too much... I mean I was there to get some help. I was not expecting advice, but some feed-back. Once she told me to think the other way round, but I would have needed to hear more things like that." (Woman)

The two men who were less satisfied with the intervention mentioned that the psychotherapist gave no advice.

Despite these criticisms, all the counsellees stated that they would recommend the method to a friend. However, 11 expressed reservations, e.g. "but not wait as long as I did", "but not with the same counsellor as I had", or "but you should not have too severe problems". The counsellees differentiated between the general benefits of the method and individual shortcomings and failures experienced.

Although this group was doubtful, less satisfied or dissatisfied with the intervention, eight of them, four women and four men, could make use of the sessions afterwards. Likewise nine women and one man mentioned that they gained new insight and ability to see problems from new angles.

#### Number of sessions

A majority of the counsellees, 36 women and five men, had four meetings with their psychotherapist during approximately one month, seven had three sessions and three had fewer.

### The telephone follow-up interview (N = 19)

A majority of the counsellees, seven women and three men, followed up by telephone were dissatisfied or doubtful about the sessions, while six women and three men were satisfied or very satisfied. In contrast, all except one stated that they would recommend the method to a friend. Those who were dissatisfied expressed mainly the same areas as the dissatisfied group among the personally interviewed: no good contact with the counsellor, four sessions were not enough. Among the satisfied counsellees, the most common statements about what they appreciated were: "talking things out", "having somebody listen", "getting advice, practical guidance".

#### Number of sessions

Of the 19 persons, four had four sessions and eight had three. The rest had one or two.

## Discussion

### Key findings

#### Self-rated mental and general health in the personally-interviewed group

The GHQ 12 (N = 42) screenings showed a halving of symptoms at group level between the two occasions. A similar improvement is reported by Mathers et al. [19].

Compared to general population studies, a high rating of symptoms as in our study is not unusual in a young population: Humphris et al report in a cross-sectional survey among undergraduate dental students in seven European countries that 36 percent of the population showed caseness for psychological distress (morbidity) at the recommended cut-off point (>3 on GHQ) [18].

In a population study in Stockholm County Council, 35 percent of all women (N = 446) and 18 percent of all men (N = 338) aged 18 – 29 years reported caseness with the same measurements as above[25].

Similarly to the rise in mastery and control there was a significant improvement in general health self-ratings between baseline and post-test 1 and post-test 1 and post-test 2. Despite this improvement – from 3.1 to 3.7 (mean value) – the personally-interviewed group did not rate their health as high as did the 18 – 29 age-group in Statistics Sweden's annual Living Conditions Survey (mean value 4.3; N = 1100)[26].

General health status compared to that of coevals was also significantly improved. Before the intervention, 53 percent rated their health worse than that of coevals. After the intervention, however, this decreased to 27 percent. Still there is a remarkable discrepancy compared to the Living Conditions Survey where 8 percent rated their health worse than that of coevals [27]. This difference between our study group and the survey group randomised from a general population can be interpreted as a sign that our group was more vulnerable.

The self-ratings expressed a clear recovery of mental and general health plus ability to take control of one's life.

#### The personal interviews

##### New insights

The personal interviews show that about 70 percent were satisfied with the intervention. The dominating themes in this group were new insights, new approach to life including fresh patterns of thinking and ability to make use of the sessions afterwards. It is remarkable that ten members of the doubtful, less satisfied and unsatisfied group (N = 16) stated their new insights and eight of them could make use of the sessions afterwards. Unfortunately we put no direct question as to whether the counsellees had improved their ability to cope with their problems, but 35 of 51 expressed that they could use what they had learnt in the counselling.

In addition, the St. Lukas Foundation accepts practically everyone seeking the counselling. The mental problems encountered are often more complicated than those for which the counselling method is intended. Considering this, the results must be seen as good.

##### Health improvement

Both minor mental symptoms and moderate and deep depressions have a natural development curve with a peak and a subsequent decline [28]. Duration is related to personality, heredity and type of problem. Medication and social support also affect progress. We did not measure personality, social support or ongoing medical treatment; factors which might have influenced the results. However, the sessions probably did have a positive effect in the group personally interviewed since all results point to an improvement in mental and general health. Those who were not satisfied were mainly discontent with their counsellor and considered that four sessions were not enough. Perhaps their problems were more severe.

##### Long-standing problems

The largest group of counsellees stated that their problems had lasted longer than three years. With long-standing and consolidated problems, a significant improvement in four weeks is remarkable. In addition, the personal interviews emphasized that the intervention had given insight and perspective and had aided structure. Mathers, et al. [19] report a similar development. Although their largest group had had problems for more than a year, symptoms measured with the GHQ-12 were almost halved after three sessions. The authors explained that the young people were in a transitional phase where prompt help could bring about a change despite long-standing problems.

#### Methodological discussion: the personally-interviewed group versus the telephone group

##### Skewed gender distribution

In the whole study population of 74 people, the gender distribution was 80/20. This relationship corresponds relatively well to that among people seeking psychotherapy in Sweden, e.g. 80% women at the Swedish Institute of Psychotherapy. However, among the *personally-interviewed group*, there was a strikingly skewed distribution among those completing the whole intervention, with 46 women and only five men. Of the 15 men in the study population, all drop-outs, four were men, and six did not fulfil the criteria for the interview. Whether a relatively bigger male dropout was a chance phenomenon or whether it was symptomatic of St. Lukas's work with young people is a relevant question. If the dropout was other than random, the intervention needs to be better adapted for men. It is indeed possible that many men need a different kind of intervention. Even if their self-reported mental ill-health according to Swedish national surveys indicates that their symptoms on the whole are half as severe as women's of the same age [23], men probably have a greater need for some kind of intervention than their patterns of visits to psychotherapeutic institutions point to.

##### Different areas of satisfaction

Those interviewed by telephone appreciated tangible measures more than did those interviewed personally. "Having received information", "practical guidance and views" were phrases more often used by both sexes in this group. A majority of those who had personal follow-up interviews expressed the value of process-directed contributions, such as "getting insight", "getting (new) perspectives", "viewing a problem from another angle". This difference can be seen from the answers to the introductory personal questionnaire, in which those who did not meet the criteria for the follow-up interview tended to have more concrete expectations of the counselling than those interviewed personally. In addition the problems of the former group had lasted a shorter time than those of the latter.

The differing appreciation of the counselling method was related to the social positions of the counsellees. More of the group interviewed by telephone worked in the service sector and only one, a woman, was an academic. It thus appears that those with an academic background are more attracted to a process-and-developmental perspective, while shorter educated, or vocationally trained, groups may be more interested in practical information and results. These tendencies do allow the hypothesis that the method suits those with higher education better than those with lower. It is also probable that the psychotherapist's social background is of importance.

Seven women and three men of 19 interviewed by telephone reported that the intervention did not live up to their expectations (negative reply to "Did you get what you wanted?"), while only 20 percent of those attending personal follow-up interviews gave this answer. Given the character of the group, this result is not particularly remarkable. No gender differences in the degree of satisfaction between those interviewed by telephone and those interviewed face-to-face was found.

### Limitations

This relatively small study was conducted consecutively without a control group, for which it could be criticized. The recruitment of a control group was discussed, i.e. the creation of a waiting list for the sessions. The idea was rejected first because the procedure would be against the counselling concept and secondly for ethical reasons. The telephone interview was necessitated by some counsellees' failure to return their follow-up questionnaires as an indication that the sessions were completed. The telephone interview was thus more arbitrary and was restricted to six core questions of the thirteen constituting the personal follow-up interview. The focus of the study was young people's experience of the counselling. For this reason we cannot usefully draw conclusions about the therapist's contributions.

Four-Sessions-at-Most appears to work well for the help-seeking group for which it was designed, that is, those who feel lost or have existential thoughts, people stuck in a rut or in temporary crisis. It is likely that the more difficult the problems are, the greater the risk that the counselling method will not help satisfactorily. This is supported by a review concluding that counselling seems to be most effective in milder forms of psychological disturbance [29].

Whether this counselling method is applicable with other ethnic groups cannot be answered from this study as only three participants had non-Scandinavian backgrounds. Also, those with lower education, and men, were comparatively few in this study, making an evaluation less well supported.

### How can the counselling method be improved?

#### More sessions when needed

Those with more difficult problems often need more comprehensive care, which cannot be given in just a few meetings. Bearing this in mind, it would be desirable for those in this category to be offered continued contact or some other support assuming they and the therapist agreed. The kind and extent of support must be assessed individually.

#### The gender perspective

Some attempt ought to be made to reach young men with the offer of counselling and to prevent them dropping out. St. Lukas has offered such counselling by advertising with photos of young males in the Swedish magazine *Nöjesguiden*. Perhaps callers could be offered the choice of male or female psychotherapist? Women could be offered the same options.

#### Follow-up meeting

It would be worth trying an option that included a follow-up meeting after two or three months. Barret-Kruse [30]lends support to the idea that counselling in one problem area may encourage help-seekers to make changes in other areas. A follow-up meeting is appropriate to further such changes and to give feedback to psychotherapists on their performance.

#### Overhaul of payment routines

Some counsellees were irritated or even insulted, especially if they were very shaken, by the payment routine whereby they handed over the fee to the psychotherapist at the end of the meeting. For longer psychotherapeutic treatment the payment from the patient is part of the treatment situation, a ritual illustrating reciprocity between the parties. But in counselling, where the aim is to start a process of change through a few problem-focused meetings, a more neutral way of paying ought to be applied.

### Policy recommendations

We urgently need to implement counselling that is easy to access. It should take place outside the psychiatric care service and in surroundings familiar to young people. Today, in Sweden, young people can obtain mental support at youth centres, student health centres and primary-maternity (for women) and child-care clinics. This support is however insufficient, less systematic and poorly funded. In Stockholm, the student care service offers e.g. a maximum of three meetings, and waiting times are often long. It is important to strengthen resources in existing fields of activity and to review the scope for short-term support in other environments e.g. employment offices. A build-up of counselling competence in such fields would probably benefit from a preventive point of view, both in human as well as economic terms, and would be well worth the cost.

### Further research

There is a need for further studies of the effectiveness with short-term counselling interventions for young adults with minor mental health problems. Changes in current society have much affected the economic, occupational and social situation for young people. We realized that special problems exist in reaching and providing a good intervention for young men, groups with short education and people with different ethnic backgrounds. It is a challenge to develop practical intervention methods addressing minor mental health problems in different groups. For these groups, other kinds of method than individual counselling should also be investigated.

## Conclusion

- The evaluation of Four-sessions-at-most showed a clear improvement in self-rated mental and general health, mastery and control in the group completing the study agreement.

- The intervention is easily accessible, economically reasonable, appreciated and probably effective for young adults with minor mental health problems.

- Due to the skewed gender distribution it is unclear if the method is appropriate for men. As in every activity, continuous internal quality development and the adoption of a gender perspective are important factors.

- It is important to focus on the social aspects of this form of short-term support and to implement it in environments familiar to young adults.

## Abbreviations

GBP = British Pound.

## Competing interests

The author(s) declare that they have no competing interests.

## Authors' contributions

RW designed and conducted the study. AB-B supervised the analysis and contributed to the interpretation of the findings. Both authors read and approved the final manuscript.

## Pre-publication history

The pre-publication history for this paper can be accessed here:


